# Giant moving vortex mass in thick magnetic nanodots

**DOI:** 10.1038/srep13881

**Published:** 2015-09-10

**Authors:** K. Y. Guslienko, G. N. Kakazei, J. Ding, X. M. Liu, A. O. Adeyeye

**Affiliations:** 1 Depto. Física de Materiales, Universidad del País Vasco, UPV/EHU, 20018 San Sebastián, Spain; 2 IKERBASQUE, the Basque Foundation for Science, 48013 Bilbao, Spain; 3 Information Storage Materials Lab., Dept. Electrical and Computer Engineering, National University of Singapore, Singapore-117576, Singapore; 4 IFIMUP and IN-Institute of Nanoscience and Nanotechnology, and Depto. Fisica e Astronomia, Universidade do Porto, 4169-007 Porto, Portugal

## Abstract

Magnetic vortex is one of the simplest topologically non-trivial textures in condensed matter physics. It is the ground state of submicron magnetic elements (dots) of different shapes: cylindrical, square etc. So far, the vast majority of the vortex dynamics studies were focused on thin dots with thickness 5–50 nm and only uniform across the thickness vortex excitation modes were observed. Here we explore the fundamental vortex mode in relatively thick (50–100 nm) dots using broadband ferromagnetic resonance and show that dimensionality increase leads to qualitatively new excitation spectra. We demonstrate that the fundamental mode frequency cannot be explained without introducing a giant vortex mass, which is a result of the vortex distortion due to interaction with spin waves. The vortex mass depends on the system geometry and is non-local because of important role of the dipolar interaction. The mass is rather small for thin dots. However, its importance increases drastically with the dot thickness increasing.

There are some fundamental conceptions in physics such as mass, charge, field etc. In the simplest case of classical Newton’s mechanics, the mass of an object (particle) is determined by its resistance to acceleration due to action of an external force, i.e., this is an inertial mass^1^. However, in general case, definition of the particle mass is not so simple because of the particle interaction with surrounding fields that essentially renormalizes the particle physical properties. Sometimes, in magnetism it is possible to assign properties of mechanical particles such as coordinate, momentum, mass etc. to an inhomogeneous magnetization texture. This approach was effectively used to describe dynamical behavior of magnetic topological solitons^2^ - domain walls, vortices and skyrmions. Below we consider a new mechanism of formation of the inertial magnetic vortex mass in a ferromagnetic dot due to interaction with spin waves. In this case, the vortex mass is a proportionality coefficient between the moving vortex energy and its squared velocity and reflects the energy increase due to the vortex dynamic profile deformations.

Usually the mass in magnetism is introduced by analogy to the effective mass of Bloch electrons in a lattice potential assuming quadratic dispersion relation for spin waves (magnons)^3^: 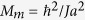, where *J* is the exchange integral, *a* is the lattice period. The value of the Bloch mass *M*_*m*_ is very small, about of 10^−30^ g. More realistic understanding of the mass having absolutely other sense was suggested by Döring^4^ to describe domain wall motion in bulk magnets. It reflects an influence of deformations of a moving domain wall on its energy (i.e., how this energy depends on velocity). The necessity of magnetic vortex mass and corresponding vortex frequency re-normalization was numerically obtained for a model system of easy plane 2D ferromagnet in the exchange approximation^5^.

The vortex excitations in patterned films are being studied extensively for the last decades^6^. Existence of the vortex low frequency gyrotropic mode dominated by the dipolar interaction was predicted^7^ and then it was observed experimentally by different experimental techniques^8^,^9^,^10^,^11^. More recently, an ultimate effect in the magnetic vortex dynamics - the vortex core polarity reversal was detected in patterned magnetic nanostructures increasing the driving force strength^12^,^13^. In this case, the moving vortex deformation leads to appearance of a dependence of the vortex energy on its velocity and eventually to the vortex core reversal following by release of the accumulated energy via emission of radial spin waves. Then, it was proven experimentally that in thick dots other kind of the vortex dynamical deformations, flexure oscillations of the vortex core string with *n* nodes along the dot thickness, can exist^14^,^15^. Very recent X-ray imaging experiments on the gyrotropic bubble domain dynamics in CoB/Pt dots^16^ showed importance of the mass contribution to describe the bubble low frequency excitation modes. The estimated mass was found to be essentially larger than the Döring mass used for calculations of the bubble domain excitation spectra within the limit of ultra-thin domain wall in Ref. 17. It was also shown that the rigid vortex model^18^ leads to essential underestimation of the mass and more adequate approach accounting for the spin wave spectra is needed^19^, especially for thick dots. Importance of the vortex - azimuthal spin waves interaction was underlined in Ref. 20, where the frequency splitting of the azimuthal spin waves was measured. We show below that neither Bloch mass nor Döring mass is sufficient to describe the GHz dynamics of topological magnetic solitons - vortices and bubble-skyrmions in restricted geometry. Some generalization of the mass accounting for additional spin degrees of freedom (spin waves) and their interaction with moving magnetic soliton is necessary.

In this study, we report broadband ferromagnetic resonance measurements and calculations of the fundamental vortex gyrotropic mode in relatively thick cylindrical permalloy (Ni_80_Fe_20_ alloy) dots with thickness 50–100 nm and radius of 150 nm. We show that the frequency of this low-frequency mode can be explained introducing an inertia (mass) term to the vortex equation of motion. The mass is anomalously large and reflects moving vortex interaction with spin waves of the azimuthal symmetry.

## Results

### Experimental design

**Figure 1:**
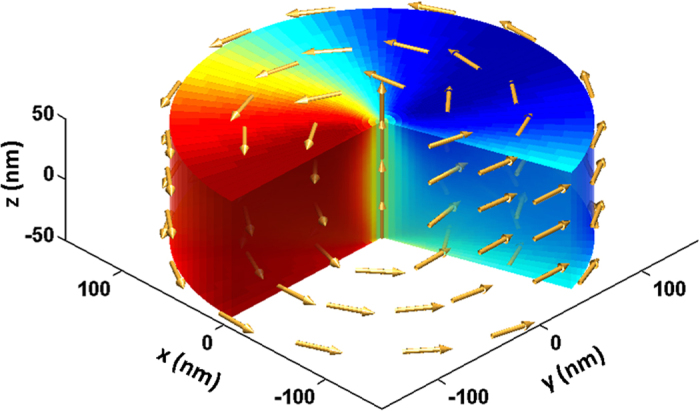
Cylindrical magnetic dot in the vortex state and the system of coordinates used. Arrows mark the local magnetization vectors in the static state. The x-component of the reduced magnetization **m** varies from +1 (deep red color) to −1 (deep blue color).

**Figure 2:**
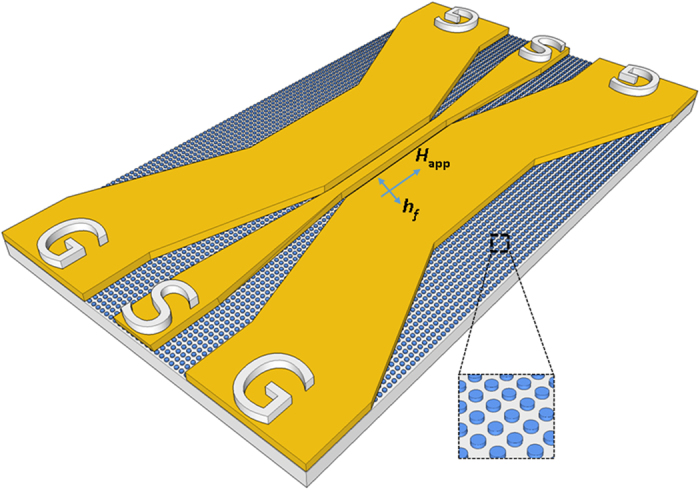
Experimental set up for high frequency measurements of the dot arrays used for detection of the fundamental vortex gyrotropic mode. The patterned film is a square array of permalloy cylindrical dots with the radius 150 nm and variable thickness in the range 40–100 nm.

Periodic two dimensional arrays of circular permalloy (Ni_80_Fe_20_) vortex state circular dots with the thickness *L* = 40–100 nm, radius *R* = 150 nm and pitch *p* = 620 nm were fabricated on Si substrates over 4 mm × 4 mm area using deep ultraviolet lithography followed by electron beam evaporation and lift-off process. Fabrication details can be found elsewhere^14^,^15^. The simulated vortex magnetization configuration is shown in Fig. 1. Axes *x* and *y* of the Cartesian coordinate system are lying in the dot array plane along square lattice diagonals (Fig. 2) and axis *z* is aligned along the dot thickness (Fig. 1). Since the distance between the dot centres is more than twice the dot diameter, interdot dipolar interactions are considered to be negligibly small.

### Microwave spectra measurements and simulations

**Figure 3:**
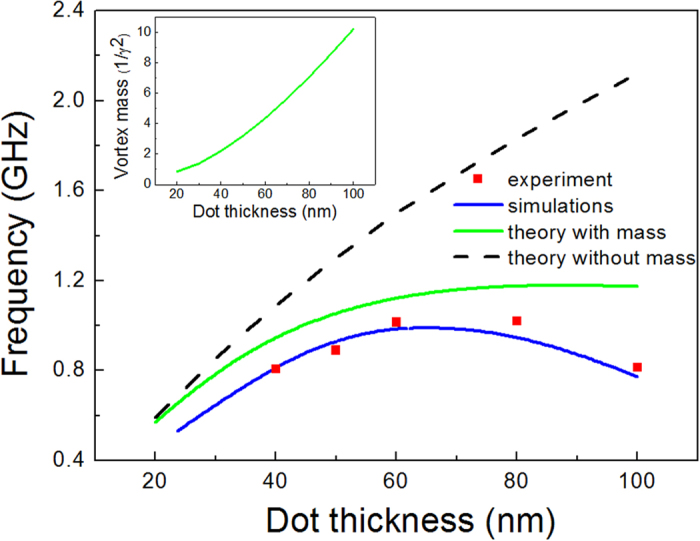
The frequency of the lowest vortex gyrotropic mode vs. dot thickness, *ω*_0_(*L*) 2p: red squares – the experimental data, blue solid line – the simulated frequencies, green solid line – the calculations according to Eq. (4) accounting vortex mass, black dashed line – calculations without accounting for the vortex mass. Inset: the dependence of the vortex mass density 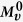 **on the dot thickness calculated by using Eq.** (9).

The microwave absorption of the dot arrays was probed using a vector network analyzer by sweeping the frequency in 50 MHz −6 GHz range in the absence of an external magnetic field at room temperature. The microwave field, *h*_rf_, is oscillating in the patterned film plane perpendicularly to the central waveguide (Fig. 2). The measured microwave excitation spectra are quite complicated. Therefore, we concentrated our attention on the lowest resonance peak that was clearly observed in the vicinity of 1 GHz. This peak was interpreted as the vortex gyrotropic mode, which is almost uniform (i.e., its dynamical magnetization profile has no nodes) along the dot thickness^14^,^15^. A careful measurements of the dependence of resonance frequency of this mode on the dot thickness demonstrate a clear maximum around the dot thickness *L* = 70 nm (see Fig. 3).

The experimental results were compared with the simulated microwave absorption spectra for the dots with dimensions identical to the experimental ones, obtained by applying a pulse excitation scheme (see Methods). As observed, the simulated resonance frequencies *ω*_0_(*L*) (Fig. 3) of the fundamental vortex mode varying the dot thickness *L* are in a very good agreement with the experimental data, demonstrating the similar maximum of the dependence *ω*_0_(*L*). From the other side, our simulations are in qualitative agreement with the simulations by Boust *et al.*^21^,^22^ for the dots of small radius *R* = 80 nm. This allows us to consider the conducted micromagnetic simulations as a reliable tool to study in details the observed vortex excitation modes in thick dots.

Simulations confirmed the assumption that the observed peaks around 1 GHz correspond to the lowest mode (no nodes along dot thickness) of the vortex gyrotropic excitation spectra (see Refs 14, 15 for detailed description of the modes). The dynamical magnetization distribution of this mode was found to be almost homogeneous at smaller thickness. However, it reveals a smooth dependence on the thickness coordinate for larger dot thickness with a minimum in the dot centre^14^,^21^.

### Analytical calculations of the vortex excitation spectra

The calculations conducted on the basis of existing analytical theory of the vortex gyrotropic mode^6^,^7^ showed that the calculated fundamental frequency *ω*_0_(*L*) is in two times larger than the experimental one for dot thickness of 80–100 nm. Accounting for the inhomogeneity of the dynamical magnetization along the dot thickness yields corrections of about 10% and, therefore, is not sufficient to explain this discrepancy. We developed a new approach to the problem introducing the magnetic vortex mass as a result of the interaction with spin waves and calculated giant values of the mass for thick dots, which can explain our measurements.

To calculate magnetization dynamics we start from the Landau-Lifshitz equation of motion 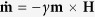 of the reduced magnetization **m** = **M**/*M*_*s*_, 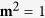
^2^. Here **H** = −*δw*/*δ***M**, *w* is the magnetic energy density 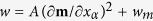, *w*_*m*_ = −*M*_*s*_**m · H**_*m*_/2 is the magnetostatic energy density, *A* is the exchange stiffness, **H**_*m*_ is the magnetostatic field, *γ* is the gyromagnetic ratio, and *x*_α_ = *x, y, z*. We distinguish two subsystems in the magnetic dot: slowly moving vortex + fast magnetization oscillations - spin waves (SW) and express magnetization as a sum 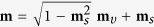 of the vortex (υ) and SW (s) orthogonal contributions, **m**_*υ*_ · **m**_*s*_ = 0. The components of **m**_*s*_ are the simplest in a moving coordinate frame *x*′*y*′*z*′, 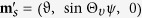, where the axis *Oz*′ is directed along the instant local direction of **m**_*υ*_ defined by the spherical angles of **m**_*υ*_(Θ_*υ*_, Φ_*υ*_) (see Methods). We consider SW magnetization **m**_*s*_ as a small perturbation of the moving vortex **m**_*υ*_ background and calculate how the SW dynamics influence the vortex dynamics.

To consider thickness dependent vortex excitations we assume that vortex magnetization can be written as **M**_*υ*_(**r**, *t*) = **M**_*υ*_(**ρ**, **X**(*z*, *t*)), where **X**(*X*, *Y*) a position of the vortex core center. Then, we can rewrite the vortex equation of motion in the Thiele form^23^ as equation for **X**:




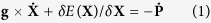




where 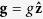, *g* = 2*πM*_*s*_/*γ* is the gyrovector density, *E* is the total magnetic energy per unit dot thickness, and 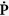 is an extra force due to the spin-wave momentum **P**(*ϑ*) (see Methods). The equation of motion (1) describes the vortex gyrotropic motion in a confining potential *E*(**X**) influenced by SW via the term 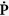 that gives an additional contribution to the magnetic energy as 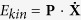.

The equations of motion for the SW variables *ϑ*(**r**, *t*), *ψ*(**r**, *t*) (neglecting the exchange interaction because *R* ≫ *L*_*e*_, 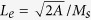 is the exchange length) are 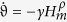, 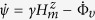, where the dynamic magnetostatic field **H**_*m*_(**r**, *t*) is defined in Methods. The equations for *ϑ*, *ψ* depend on time derivative of the moving vortex phase Φ_*υ*_(**X**). The derivative 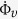 is calculated within the two vortex model^6^,^7^ as 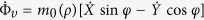, where 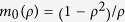 is the radial profile of the vortex gyrotropic mode^24^, *ρ* is in units of *R*. We use the cylindrical coordinates **r** = (*ρ*, *φ*, *z*). Substituting the solution of inhomogeneous equation 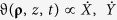 to the vortex-SW interaction Lagrangian per unit thickness 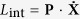 (see Methods) we can write it as a vortex kinetic energy




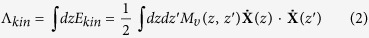




where *M*_*υ*_(*z*, *z*′) is the nonlocal vortex mass density, see Methods. The mass term (2) reflects dependence of the moving vortex energy on its velocity and appears due to a vortex structure deformation resulting from hybridization with high-frequency azimuthal spin waves.

The equation of motion (1) of the vortex core position **X** can be written accounting the mass term (2) as (see Methods)




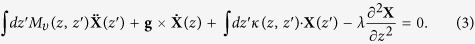




Solution of Eq. (3) leads to renormalization of the massless vortex gyrotropic frequencies. The eigenfrequency of the *n*-th gyrotropic mode is




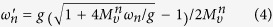




where *ω*_*n*_ is the eigenfrequency of bare, massless vortex, and 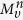 is the diagonal component of the vortex mass density (see Methods, Eq. (9)).

## Discussion

The finite vortex mass density 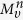 gives always negative contribution to the vortex gyrotropic eigenfrequencies given by Eq. (4). The calculations conducted using Eq. (9) showed that the mass density increases with the dot thickness increasing (see Fig. 3) and sharply decreases with the gyrotropic mode number *n* increasing. The non-monotonous dependence of the fundamental vortex gyrotropic mode frequency on the dot thickness similar to shown in Fig. 3 was simulated by Boust *et al.*^21^ without explanation of its origin. The mass is of principal importance for explanation of the fundamental vortex frequency (*n* = 0) leading to the gyrotropic frequency decrease in 2 times for the dot thickness *L* = 80–100 nm (Fig. 3). There is a smooth maximum on the calculated dependence 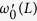 at the dot thickness *L* = 80 nm. Whereas, the experimental and simulated maxima of the dependence 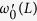 are more pronounced. *I.e.*, the mass density 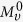 is higher than the calculated one using Eq. (9). Accounting for the experimental value 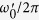 = 0.83 GHz we get for the fundamental vortex gyrotropic mode mass the giant value of 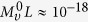 g for the dot thickness *L* = 100 nm. This mass is in 11–12 orders of magnitude larger than the typical magnon mass 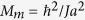, in two orders of magnitude larger than the vortex domain wall mass 6.2 10^−21^ g measured by Bedau *et al.*^25^,^26^ and in 3 orders of magnitude larger than a typical Döring mass of quasi 1D-domain walls^4^. The vortex mass is comparable with the bubble-skyrmion mass estimated recently as >8 10^−19^ g by Büttner *et al.*^16^. The vortex mass is giant (especially, in comparison with the Bloch mass) because it is proportional to degree of complexity of the spin texture, the number of spin deviations from the aligned spin state. These deviated spins are mainly located in the vortex core, and their number increases by increasing the dot thickness. There is just one reversed spin for the Bloch magnon and the corresponding effective mass is small.

The calculated vortex mass is principally different from the effective gyrotropic mass, *m*_*G*_ ≈ 10^−19^ g, introduced formally by Wysin *et al.*^27^ on the basis of the Thiele equation. The mass *m*_*G*_ is proportional to the dot radius and is thickness independent. Whereas, the mass calculated as result of the vortex-SW interaction, 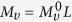, increases strongly with *L* increasing and weakly depends on *R*. Small values of the vortex mass density 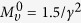 and 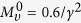 calculated^19^ and simulated^28^ previously in the limit of thin dots *L*/*R* ≪ 1 are in agreement with the present calculations of 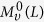 for the dot thickness *L* ≈ 20 nm.

The central point of our model of the magnetization dynamics is the vortex-SW dynamical interaction (see Eq. (5) and details in Methods). Therefore, a question arises: are there any experimental or simulation evidences that such interaction does exist? It was established experimentally that the vortex motion influences the azimuthal spin waves resulting in a splitting of their frequencies for the spin wave modes with indices *m* = +1/−1^20,29,30^. Moreover, it was shown experimentally and by simulations in the papers^29^,^30^ that removing of the vortex core results in disappearance of the splitting of the azimuthal mode frequencies. And vice versa, it was demonstrated by X-ray microscopy and by simulations that exciting the azimuthal spin waves it was possible to excite the vortex core motion, increase its magnitude up to the vortex core polarization reversal^31^. *I.e.*, there is no doubts that such vortex-SW interaction exists.

The introduced mechanism of magnetic vortex mass formation via interaction with the azimuthal spin waves is similar to appearance of the mass of some elementary particles via the Higgs mechanism (interaction with the Higgs field)^32^,^33^. In our case, the azimuthal magnons play a role of the Higgs bosons (excitations of the Higgs field). The geometrical gauge field (represented by the vortex variables) acquires a finite mass due to coupling with the magnons. This mass can be written in terms of the moving vortex mass within the Thiele approach to the magnetic vortex motion. Recent simulations^34^ showed that the drop of domain wall mobility at high velocity in a magnetic nanotube can be interpreted as increasing of the wall mass due to emitting of spin waves. This is an additional confirmation that appearance of the dynamical magnetic soliton (domain wall, vortex, skyrmion) mass is a general effect which might be observed in many magnetic patterned nanostructures including circular magnetic dots, nanotubes, nanostripes etc.

Summarizing, we found by broadband ferromagnetic resonance measurements that in ferromagnetic nanodots, the eigenfrequency of the fundamental vortex gyrotropic mode reveals a maximum as function of the dot thickness. The frequency of this mode is calculated by introducing an inertia (mass) term to the vortex equation of motion. The mass is anomalously large and reflects moving vortex interaction with traveling spin waves of the azimuthal symmetry. The mass is non-local due to non-locality of the magnetostatic interaction. The observed behaviour is explained on the basis of developed analytical theory and confirmed by micromagnetic simulations.

## Methods

### Micromagnetic simulations

Frequency and spatial distribution of the observed vortex modes were obtained from the micromagnetic simulations that were performed using commercial LLG code^35^. Standard parameters for Ni_80_Fe_20_ (exchange constant *A* = 1.05 × 10^−6^ erg·cm^−1^, gyromagnetic ratio *γ* = 2.93 ×2p GHz/kOe and anisotropy constant *K*_*u*_ = 0) were used. The values of saturation magnetization *M*_*s*_ = 810 emu/cm^3^ and Gilbert damping parameter *α* = 0.01 were extracted from ferromagnetic resonance measurements on a reference 60 nm thick Ni_80_Fe_20_ continuous film. Cell size was fixed at 5 nm ×5 nm ×5 nm. The dot thickness *L* was varied in the range 20–100 nm. The simulations were carried out for the individual dots because the interdot distances in the measured dot arrays were large enough to neglect the dipolar interdot interactions. To reveal the microwave absorption spectra in the broad frequency range a short dc magnetic field pulse with duration of 50 ps and amplitude of 50 Oe was applied along the *x* axis. The spatial characteristics of the different excited vortex modes were quantified using spatially and frequency-resolved fast Fourier transform imaging^14^,^15^.

### Analytical calculations of the vortex mass

To describe magnetization dynamics we use the Lagrangian




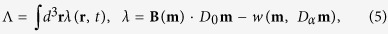




where 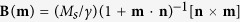, ***n*** is an arbitrary unit vector^19^, and 
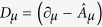
. The gauge vector potential 
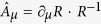
 is determined by the rotation matrix *R*(Θ_*υ*_, Φ_*υ*_) from the initial *xyz* coordinate frame to *x*′*y*′*z*′ frame (the index *μ* = 0, 1, 2, 3 denotes the time and space coordinates *x*_μ_ = *t, x, y, z* and ∂_*μ*_ = /∂*x*_*μ*_). The operator 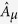 acts on the SW magnetization and can be represented by the time- and spatial derivatives of the vortex angles (Θ_*υ*_, Φ_*υ*_).

The Lagrangian (Eq. (5)) then can be re-written in the form Λ = Λ_*υ*_ + Λ_*sw*_ + Λ_int_, where 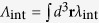 is the interaction term between the moving vortex magnetization **m**_*υ*_ described by the Lagrangian 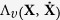 and spin waves, which are described by the Lagrangian density 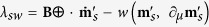. The magnetization **m**(**r**, *t*) is expressed via the angles Θ(**r**, *t*) = Θ_*υ*_(**r**, *t*) + *ϑ*(**r**, *t*), Φ(**r**, *t*) = Φ_*υ*_(**r**, *t*) + *ψ*(**r**, *t*), where 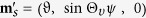 describes SW excitations, **r** = (**ρ**, *z*), **ρ** is the in-plane radius vector, and *z* is the thickness coordinate. The 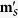-components are used in the form *ϑ*(**ρ**, *z*, *t*) = *a*_*v*_(*ρ*, *z*)cos(*mφ* − *ωt*), *ψ*(**ρ**, *z*, *t*) = *b*_*v*_(*ρ*, *z*)sin(*mφ* − *ωt*), where *a*_*ν*_*, b*_*ν*_ are the SW amplitudes, *v* = (*n*, *m*, *l*) (*n* = 0, 1, 2 …, *m* = 0, ±1, ±2, …, *l* = 0, 1, 2 …) *n* and *l* is number of the nodes of dynamical SW magnetization along thickness and radial directions, respectively. The dynamic vortex-SW coupling induced by the component 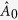 exists only for the azimuthal modes with *m* = ±1. The SW and interaction Lagrangian density are 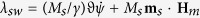 and 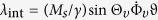, correspondingly. Here 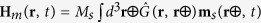 is the dynamic magnetostatic field, the kernel 
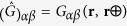
 is the magnetostatic tensor^23^, *α, β* = *ρ*, *φ*, *z*. The spin-wave field momentum 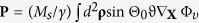 corresponding to *λ*_int_ determines the vortex-SW interaction Lagrangian 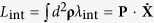.

The spin wave eigenfrequencies/eigenfunctions can be found from solution of the linear integral equation in the limit *F*(*η*) → 0:




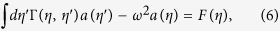




where the integral kernel is 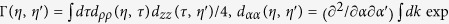 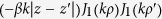 are the magnetostatic Green functions, *α*, *α*′ = *ρ*, *z* and the function *F*(η) describing the SW-vortex interaction is 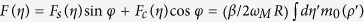

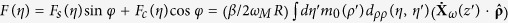
, *η* = (*ρ*, *z*), *dη* = *ρdρdz*, the frequency ω is in units of ω_*M*_ = *γ*4*πM*_*s*_.

For the SW variables *ϑ* and *ψ* without interaction with the vortex core (*F*(*η*)=0), we reduce the problem to eigenvalue problem for the integral magnetostatic operator and get a discrete set of magnetostatic eigenfunctions **μ**_*v*_(**r**) and corresponding SW eigenfrequencies *ω*_*v*_(*v* = (*n*, *m*, *l*)), which are well above the fundamental gyrotropic eigenfrequency, *ω*_0_. The solution of inhomogeneous equation (6) *ϑ*(**ρ**, *z*, *ω*) can be represented by the resolventa 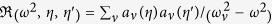.

Introducing the complex variable *s* = *s*_*x*_+*is*_*y*_ for the dimensionless vortex core position **s** = **X**/R and performing the Fourier transform *s*(*z*, *t*) = *s*(*z*)exp(*iωt*) Eq. (3) can be written as an integro-differential equation for *s*(*z*) with a nonlocal potential^14^:




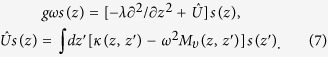




We assume that the eigenfunctions of Eq. (7) are decomposed in the series of cos *q*_*n*_*z* (*q*_*n*_=*nπ*/*L* to satisfy the dot face boundary conditions). Then, for finding the eigenfrequencies we use diagonal approximation (*n* *=* *n*′) in the matrix equation (7) and define the diagonal matrix elements of the vortex mass (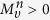) per unit dot thickness as 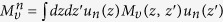, 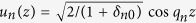.

The nonlocal vortex mass density 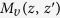 is calculated as




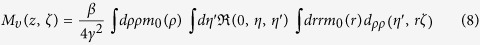




The mass density can be written as a separable kernel using explicit summation over the azimuthal spin wave spectra




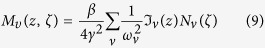




where 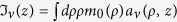 is the overlapping integral of the vortex gyrotropic mode *m*_0_(*ρ*) and the unperturbed SW eigenmode *g*_*v*_(*ρ*, *z*) obtained from solution of homogeneous Eq. (6), numbered by the index ν and normalized to unit, 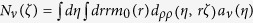 describes dipolar interaction between the moving vortex and azimuthal spin waves, and *ω*_*v*_ are the eigenvalues of homogeneous Eq. (6). We accounted that the lowest gyrotropic eigenfrequency is essentially smaller than the SW frequencies, *ω*_0_ ≪ *ω*_*v*_. The diagonal component 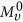 of the mass density corresponding the eigenfrequency *ω*_0_ calculated by using Eq. (9) is approximately equal to 10/*γ*^2^ for *L* = 80–100 nm, *R* = 150 nm, whereas the value of **≈**0.5/*γ*^2^ was calculated in Ref. 18 for the same dot sizes within the rigid vortex model. The components of the vortex mass density 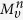 corresponding to the high-order vortex gyrotropic modes 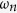 (*n* ≥ 1)^14^ are essentially smaller and result only in a small renormalization of the eigenfrequencies *ω*_*n*_.

The energy *E* in Eq. (1) can be calculated as sum of the magnetostatic and exchange energy using the definitions, 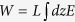, and




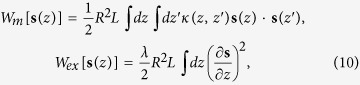




where 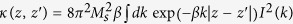, 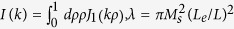 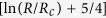 is the exchange stiffness coefficient perpendicular to the dot plane, *β* = *L*/*R*, and *R*_*c*_(*L*) is the vortex core radius^6^.

## Additional Information

**How to cite this article**: Guslienko, K. Y. *et al.* Giant moving vortex mass in thick magnetic nanodots. *Sci. Rep.* **5**, 13881; doi: 10.1038/srep13881 (2015).
